# Revealing the Functions of the Transketolase Enzyme Isoforms in *Rhodopseudomonas palustris *Using a Systems Biology Approach

**DOI:** 10.1371/journal.pone.0028329

**Published:** 2011-12-08

**Authors:** Chia-Wei Hu, Ya-Ling Chang, Shiang Jiuun Chen, Ling-Long Kuo-Huang, James C. Liao, Hsuan-Cheng Huang, Hsueh-Fen Juan

**Affiliations:** 1 Institute of Molecular and Cellular Biology, National Taiwan University, Taipei, Taiwan; 2 Department of Life Science, National Taiwan University, Taipei, Taiwan; 3 Institute of Ecology and Evolutionary Biology, National Taiwan University, Taipei, Taiwan; 4 Department of Chemical and Biomolecular Engineering, University of California, Los Angeles, CA, United States of America; 5 Institute of Biomedical Informatics and Center for Systems and Synthetic Biology, National Yang-Ming University, Taipei, Taiwan; Laurentian University, Canada

## Abstract

**Background:**

*Rhodopseudomonas palustris (R. palustris)* is a purple non-sulfur anoxygenic phototrophic bacterium that belongs to the class of proteobacteria. It is capable of absorbing atmospheric carbon dioxide and converting it to biomass via the process of photosynthesis and the Calvin–Benson–Bassham (CBB) cycle. Transketolase is a key enzyme involved in the CBB cycle. Here, we reveal the functions of transketolase isoforms I and II in *R. palustris* using a systems biology approach.

**Methodology/Principal Findings:**

By measuring growth ability, we found that transketolase could enhance the autotrophic growth and biomass production of *R. palustris*. Microarray and real-time quantitative PCR revealed that transketolase isoforms I and II were involved in different carbon metabolic pathways. In addition, immunogold staining demonstrated that the two transketolase isoforms had different spatial localizations: transketolase I was primarily associated with the intracytoplasmic membrane (ICM) but transketolase II was mostly distributed in the cytoplasm. Comparative proteomic analysis and network construction of transketolase over-expression and negative control (NC) strains revealed that protein folding, transcriptional regulation, amino acid transport and CBB cycle-associated carbon metabolism were enriched in the transketolase I over-expressed strain. In contrast, ATP synthesis, carbohydrate transport, glycolysis-associated carbon metabolism and CBB cycle-associated carbon metabolism were enriched in the transketolase II over-expressed strain. Furthermore, ATP synthesis assays showed a significant increase in ATP synthesis in the transketolase II over-expressed strain. A PEPCK activity assay showed that PEPCK activity was higher in transketolase over-expressed strains than in the negative control strain.

**Conclusions/Significance:**

Taken together, our results indicate that the two isoforms of transketolase in *R. palustris* could affect photoautotrophic growth through both common and divergent metabolic mechanisms.

## Introduction

Systems biology is a relatively new field that aims at a system-level understanding of biological systems. Recent progress in the field of molecular biology has enabled enormous amounts of data to be obtained [1] and, with the advent of high-throughput proteomics and microarray technologies, the study of systems biology has become possible [2], [3]. The microarray technique is a powerful, high-throughput, functional genomics method for accurately determining changes in global gene expression [4], [5]. In proteomics, powerful high-throughput methods allow the study of the complete set of proteins (the proteome) that are expressed at a given time in a cell, tissue, organ or organism [6].

*Rhodopseudomonas palustris* (*R. palustris*) is a purple nonsulfur anoxygenic phototrophic bacterium that belongs to the α-proteobacteria class. It is a common soil and water bacterium that lives by converting sunlight to energy and by absorbing atmospheric carbon dioxide and converting it to biomass [7]–[10]. The availability of the complete annotated genome sequence of *R. palustris* and the shotgun proteomics data of four different metabolic pathways serves as a powerful platform for more detailed systems biology characterizations [8], [11].

Photoautotrophism is one of the major pathways by which autotrophic bacteria assimilate CO_2_. In photoautotrophic conditions, the organic carbon source that is necessary to sustain metabolic requirements in autotrophic organisms can be synthesized from inorganic carbon sources through CO_2_ fixation. In most autotrophic bacteria, the Calvin-Benson-Bassham (CBB) reductive pentose phosphate cycle is the primary route for CO_2_ assimilation. Under photoautotrophic conditions, photosynthesis is used as an energy generating mechanism in the CBB cycle, which not only allows the bacteria to meet their demand for carbon but also balances their redox status [12]–[16]. When facing higher redox pressures, the CBB cycle can function as an electron sink with CO_2_ as an electron acceptor [17]. Therefore, CO_2_ fixation and reduction are substantially enhanced to enable the consumption of excess or accumulated reducing equivalents [18], [19]. The proteins within the CBB cycle include transketolase I (cbbT1), transketolase II (cbbT2), phosphoribulokinase (cbbP), fructose-1,6-bisphosphate aldolase (cbbA), ribulose 1,5-bisphosphate carboxylase/oxygenase (cbbLS) and D-fructose 1,6-bisphosphatase (cbbF). Cyanobacteria have been used as the model by which to study the regulation of the catalytic enzymes involved in the Calvin cycle, with genetic engineering techniques used to enhance photosynthetic yield and growth [20]. Some studies have indicated that exogenous expression of some of these catalytic enzymes, such as cbbA and cbbF, significantly improves photosynthetic capacity and growth [20]–[22]. However, studies of transketolase I and transketolase II in anaerobic photoautotrophic bacteria have yielded inconclusive results.

Transketolase, a key enzyme involved in the reductive CBB cycle and non-oxidative part of the pentose phosphate pathway, plays a critical role in connecting the pentose phosphate pathway to glycolytic intermediates [23], [24]. In various organisms, including bacteria, plants and mammals, transketolase occurs in two or more isoforms; however, the functional and physiological differences between the various isoforms of transketolase are still unclear. In most cells, transketolase functions in the cytoplasm to facilitate the carbon flow of the pentose phosphate pathway [25]. In contrast, transketolases responsible for the Calvin cycle within the chloroplasts of plant cells were found to be localized around the stroma and attached to the thylakoid membranes, implying a possible difference in transketolase distribution and function in photosynthetic organisms such as photoautotrophic bacteria [26], [27].

**Figure 1:**
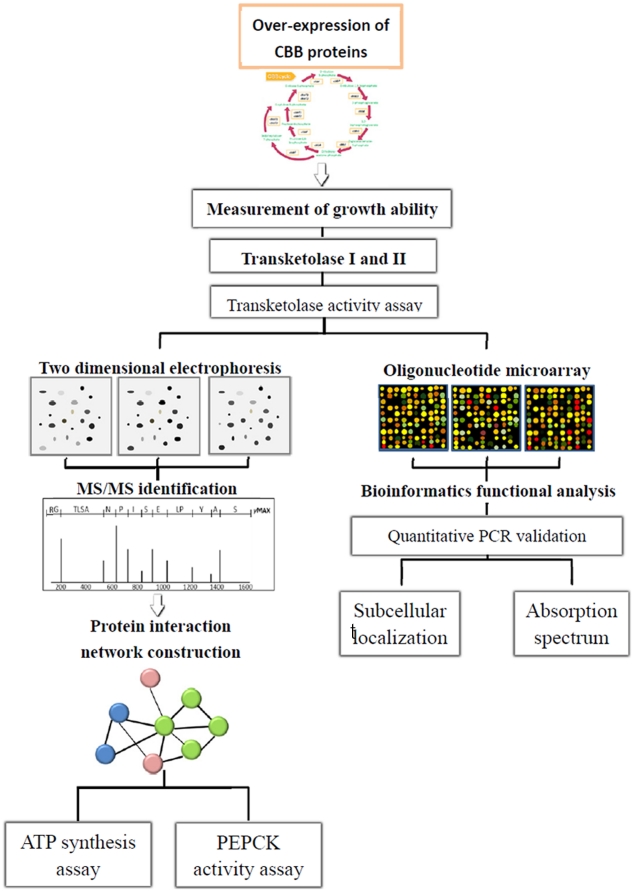
Schematic representation of the experimental design. Potential *cbb* genes that might affect autotrophic growth were constructed then assessed for their actions on growth ability. The effects of these candidates on autotrophic growth were studied in a variety of ways including measurement of their actions on enzyme activity, and observation of their subcellular localization and absorption spectra. Finally, differentially expressed proteome profiles in strains in which these genes had been overexpressed were compared to ascertain the mechanisms that might regulate autotrophic growth.

To elucidate the effects of proteins involved in the CBB cycle on the photoautotrophic growth of *R. palustrius*, the growth abilities of *R. palustrius* strains overexpressing different CBB cycle proteins were measured. We revealed that the overexpression of transketolase isoforms I and II, can contribute to cell growth; we therefore analyzed the gene and protein expression profiles of transketolase I and II using microarray assays, proteomics and functional studies. This study focuses on the contribution of transketolase isoforms to the enhancement of autotrophic growth in *R. palustris*. A diagrammatical overview of the study is given in Figure 1.

## Results

### Enhanced autotrophic growth of *R. palustris* by overexpression of transketolase

The CBB cycle plays a major role in autotrophic growth due to its participation in CO_2_ assimilation [19]. To determine the key enzyme affecting photoautotrophic growth in the CBB cycle and other regulatory systems, we overexpressed several CBB proteins, including cbbT1, cbbT2, cbbP, cbbA, cbbLS, and cbbF, by cloning each gene into *R. palustris* CGA010 gentamycine-resistant plasmids MCS-5 [28]. Seven manipulated strains with different CBB genes were produced, as described in detail in Table S1.

**Figure 2:**
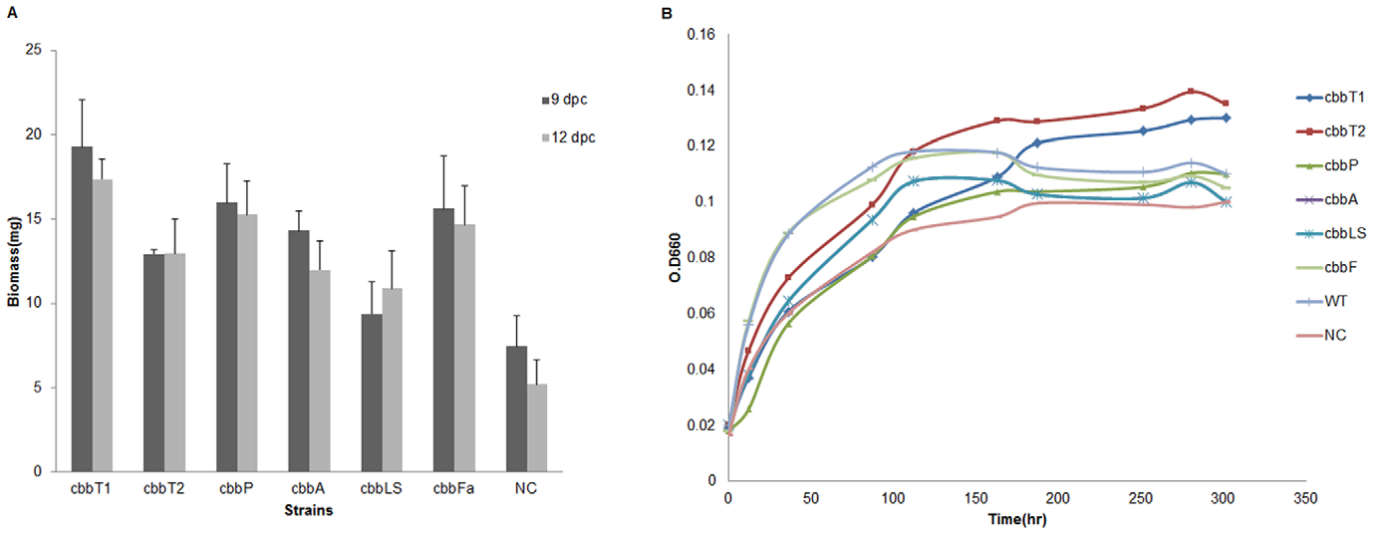
The effects of transketolase overexpression on photoautotrophic growth. (A) The effects of transketolase I and transketolase II overexpression on autotrophic growth in *R. palustris* compared with the negative control strain. Biomass analysis was performed to ascertain the CO_2_-fixing ability of the different strains. The initial number of cells in each strain culture was 10^8^. Each value represents the mean of three replicate cultures grown under identical conditions over the course of 9 days. (B) Growth curves of the transketolase-overexpressing strains of *R. palustris*. cbbT1 indicates the transketolase I-overexpressing strain; cbbT2, the transketolase II-overexpressing strain; NC, the negative control. *, *p*<0.05; **, *p*<0.005.

Under autotrophic conditions, the inorganic carbon source of CO_2_ is converted into an organic carbon source that can be utilized by the bacteria via the CBB cycle [15], [16], [29]. Consequently, enhancement of the CBB cycle or other regulatory systems would be expected to increase CO_2_ assimilation and hence increase organic carbon levels, leading to higher biomass production [30], [31]. To elucidate the effects of different CBB proteins on autotrophic growth, we investigated the differences in the growth of each manipulated strain by examining their biomass as dry cell weight (DCW) and constructed growth curves under photoautotrophic conditions. Our results showed an elevation in DCW in most manipulated strains (Figure S1). Surprisingly, the transketolase-overexpressing strain, but not the RubisCO-overexpressing strain, displayed a greater growth ability both in terms of biomass and growth curve compared with other strains (Figure S1). Transketolase-overexpressing strains exhibited increased growth under photoautotrophic conditions, indicating a crucial role for transketolase in autotrophic growth (Figures 2A and 2B).

### Physiological and functional changes arising from the overexpression of transketolase I and II in *R. palustris*

**Figure 3:**
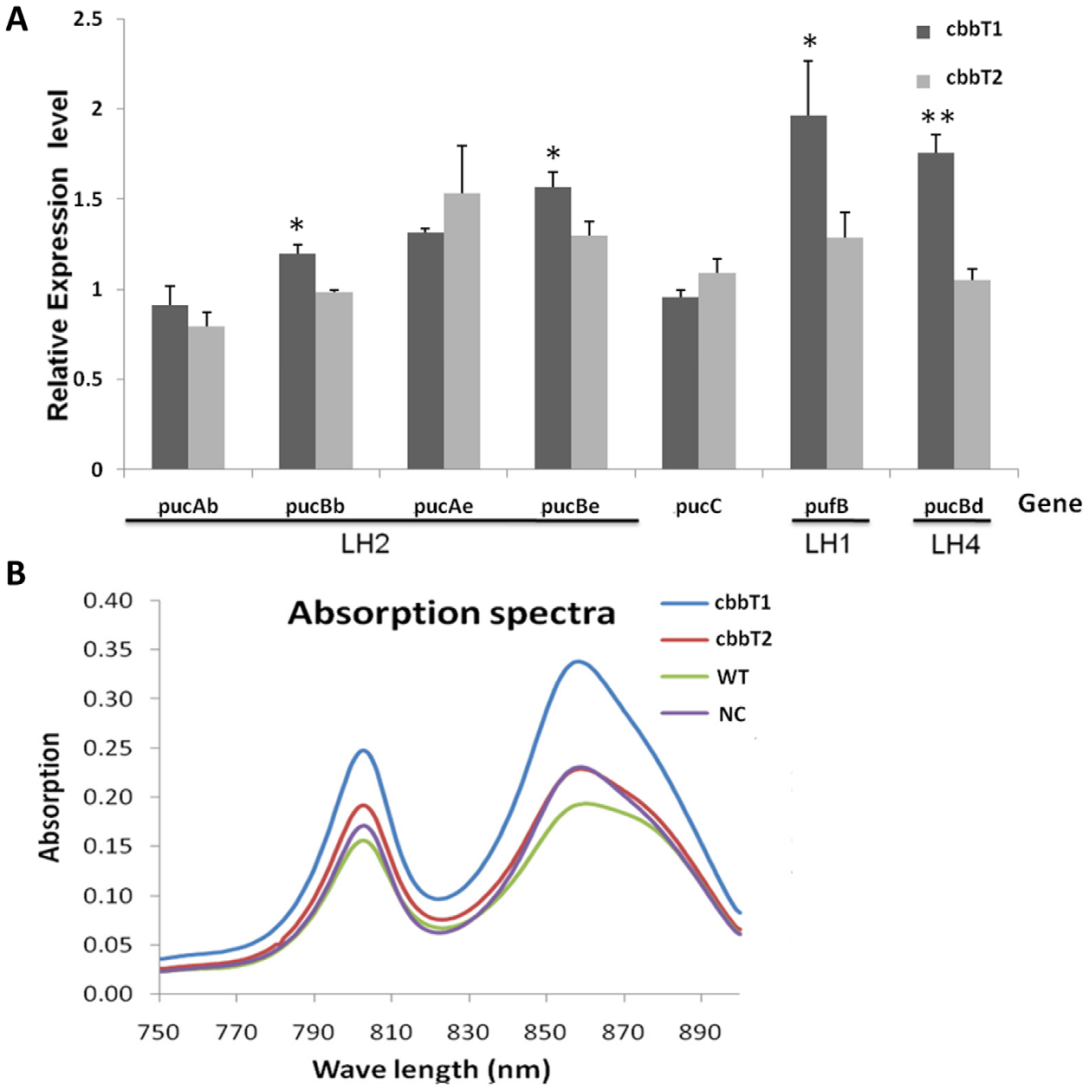
Physiological differences between the *R. palustris* strains in which overexpression of one of the two isoforms of transketolase had been induced. (A) qPCR analysis of the photosynthetic genes that encode the subunits of the light harvest complex. The ratios indicate the gene expression level of transketolase overexpression relative to negative control strain. The error bar represents SD (n = 3) between the transketolase I and transketolase II-overexpressing strains. *, *p*<0.05; **, *p*<0.001. (B) Absorption spectra of cells grown under photoautotrophic conditions. LH I absorbed at 880 nm; LH II complexes at 802 nm and 862 nm; LH IV at 802 nm.

According to our results with biomass and growth curves, the overexpression of transketolase significantly enhanced autotrophic growth in *R. palustris*. To elucidate the effects of transketolase on gene expression profile, we analyzed the transketolase-overexpressing transcriptome, and compared it with that of a negative control strain, using Agilent oligonucleotide microarray analysis. Genes showing significant changes in expression were categorized based on cellular components and biological processes they are associated with, using Blast2GO. The 51 and 11 differentially expressed genes of transketolase I- and transketolase II-overexpressing strains were analyzed against 3710 of 4820 *R. palustris* genes, selected with putative gene ontology annotation, as reference. Some categories associated with photosynthesis, including plasma membrane-derived chromatophore membrane, photosynthetic membrane, and plasma membrane light-harvest complex, were over-represented in transketolase I-overexpressing strains (Materials and Methods S1, Tables S2 and S3). These results suggest a possible difference between the functional mechanisms of the transketolase I and II isoforms. To determine whether the stimulation of the CBB cycle by transketolase I/II overexpression occurred via different pathways or physiological actions, we examined the expression of different genes associated with photosynthesis and consequent physiological differences in the transketolase-overexpressing strains. We first analyzed the relative expression levels of light harvest complex (LH) I, II and IV photosystems-related genes in both transketolase-overexpressing strains compared with negative control, with *rpoD* as an internal control transcript (Figure 3A). Genes encoding the subunits of the LH II complex, *pucBb* and *pucBe*, were significantly upregulated (*p*<0.05) in the transketolase I-overexpressing strain compared with the transketolase II-overexpressing strain. There was no significant change in the expression levels of *pucAb*, *pucAe* and *pucC* between the transketolase I and transketolase II-overexpressing strains. In addition, the relative expression levels of *pufB* (the LH I β subunit) and *pufBd* (the LH IV β subunit) were increased by 2.0- and 1.8-fold respectively in the transketolase I-overexpressing strain, but only increased by 1.3- and 1.1-fold in the transketolase II-overexpressing strain, compared with the negative control (NC) strain. The relative increases of the expression levels of these genes were consistent with the absorbance results. These results showed that the overexpression of transketolase I greatly influences the relative expression level of photosystem-related genes. The result was further confirmed by measuring photosynthesis efficiency. We isolated the intracytoplasmic membrane (ICM), in which the LH complex is located and photosynthesis occurs. The absorption spectra of transketolase I and transketolase II-overexpressing *R. palustris* strains were compared. We measured the absorption of each spectral region containing peak absorption wavelengths for the various light complexes (LH I, LH II and LH IV) [32], [33] as shown in Figure 3B. The absorption spectra of the four strains displayed similar profiles. Overexpression of transketolase I resulted in increases in the peak amplitudes of the absorption spectrum. There was no significant difference in the transketolase II-overexpressing strain compared to the control. The transketolase I-overexpressing strain showed a particularly pronounced increase at 802 nm which is the absorption peak of LH II and LH IV and 862–880 nm which is the absorption peak of LH I and LH II.

### Subcellular *in situ* localization of transketolase I and II in *R. palustris*

**Figure 4:**
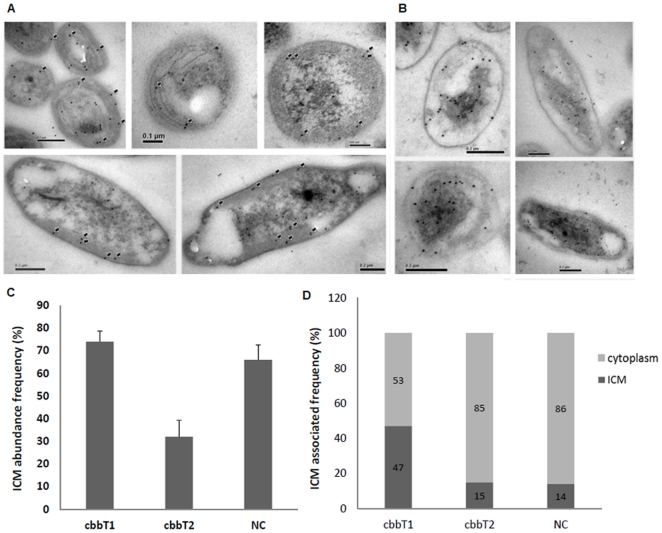
The different localizations of transketolase I (cbbT1) and II (cbbT2) in *R. palustris*. (A) The *in situ* localization of HA-CbbT1 in the cbbT1-overexpressing strain grown under photoautotrophic conditions. Localization of HA-CbbT1 was detected using 10 nm immunogold labeled anti-HA antibody in ultra-sections. (B) The *in situ* localization of HA-CbbT2 in the cbbT2-overexpressing strain grown under photoautotrophic conditions. Localization of HA-CbbT2 was detected using 10 nm immunogold-labeled anti-HA antibody in ultra-sections. (C) The ICM distributions of the transketolase isoforms in transketolase I and transketolase II-overexpressing strains. More than 300 longitudinal-section micrographs from 5–6 grids of each strain were evaluated to quantify the density of cells with significant ICM structure in cbbT1- and cbbT2-overexpressing strains. The dark arrows indicate the location of HA-CbbT1 and HA-CbbT2 in the ICMs and cytoplasm. (D) The distribution of immunogold-labeled HA-CbbT fusion protein in the ICM and cytoplasm revealed in ultrathin section micrographs of 30 different bacterial cells of the transketolase I and II-overexpressing strains and the NC strain. cbbT1 indicates transketolase I overexpression; cbbT2, transketolase II overexpression; NC, negative control.

To demonstrate the localization of transketolase I and II, cbbT1 and cbbT2 containing an epitope tag from the hemagglutinin of the human influenza A virus (HA) was constructed and overexpressed in *R. palustris*, respectively, then become HA-cbbT1/HA-cbbT2. The expression location of HA-tag fusion protein was identified by utilizing TEM. We used immunogold-labeled anti-HA antibody to probe ultrathin sections of the bacteria, which were then visualized with TEM and the percentage of bacteria with remarkably ICM structure observation and the distribution of localized HA-cbbT1/HA-cbbT2 in the overexpressed bacteria were measured. Comparing with overexpressed strains, a non-specific distribution was observed in negative control strain (Figure S2). The morphology of ICM structure and the figure of negative control strain were shown in Figure S2. As shown in Figures 4A and 4B, HA-transketolase I was mainly located within or near the ICMs, while HA-transketolase II was found mainly in the cytoplasm. It is noteworthy that the ICM structures of the transketolase II-overexpressing strain appeared to be less abundant than those of the transketolase I-overexpressing strain. As can be seen in Figure 4C, in the transketolase II-overexpressing strain, only 33% of total HA-transketolase II was located at the ICM, less than half the total HA-transketolase I seen at the ICM in the transketolase I-overexpressing strain (which amounted to 74% of total transketolase I in this strain). The distribution of total immunogold label within each bacterial section showed that HA-transketolase I was much more frequently associated with the ICM than was HA-transketolase II, which was mostly distributed in the cytoplasm (Figure 4D). Taken together, the two transketolase isoforms have different spatial localizations and therefore might have different functions in *R. palustris*.

### Protein profiles and protein interaction networks of transketolase-overexpressing *R. palustris*

**Figure 5:**
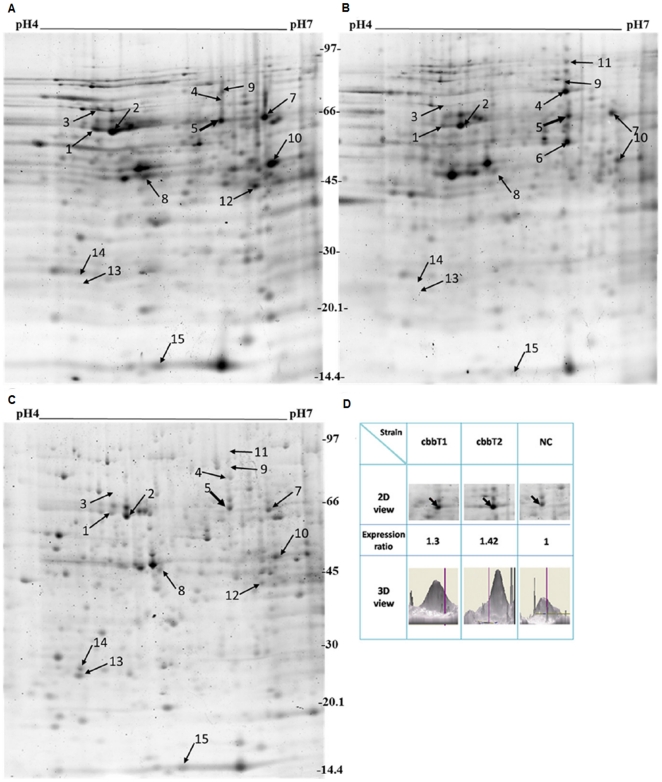
Two dimensional electrophoresis (2DE) of transketolase-overexpressing *R. palustris*. (A) 2DE of *R. palustris* CGA010 strains overexpressing transketolase I. (B) 2DE of *R. palustris* CGA010 strains overexpressing transketolase II. (C) 2DE of the negative control (NC) *R. palustris* CGA010 strains. Proteins indicated in these maps were considered to be differentially expressed and were further identified by MS. The bold arrow indicates the protein spot left by phosphoenolpyruvate carboxykinase (PEPCK). (C) 2D and 3D views of PEPCK expression levels. The expression of PEPCK was increased in transketolase I and II-overexpressing strains. Arrows indicate the spot corresponding to PEPCK. cbbT1 indicates transketolase I overexpression; cbbT2, transketolase II overexpression; NC, negative control.

To determine the possible influence of transketolase overexpression on proteins downstream to transketolase, we performed a proteomics approach based on two dimensional electrophoresis (2DE) analysis to explore differential protein expression profiles in transketolase I and II-overexpressing and negative control strains (Figure 5A to 5C, respectively). Differentially expressed protein spots were excised, digested and analyzed using mass spectrometry. We successfully identified a total of 15 differentially expressed proteins after database searching, as shown in Table S4. Proteins involved in carbon metabolism (e.g. acetate CoA ligase and phosphoenolpyruvate carboxykinase), energy production (e.g. ATP synthase subunits) and transport (e.g. branched chain amino-acid ABC transporter substrate-binding protein, extracellular solute-binding protein, family 1 and ABC transporter, periplasmic amino acid binding protein aapJ-1) exhibited upregulation in the transketolase-overexpressing strains. Upregulation of proteins associated with the transport and metabolism of carbohydrates and amino acids could explain the enhanced growth observed in the transketolase-overexpressing strains. For example, extracellular solute-binding proteins usually serve as recognition constituents of transport systems that bind to oligosaccharides or iron [34]. Another up-regulated protein, periplasmic amino acid binding protein aapJ-1, is essential for both the uptake and efflux of amino acids [35], [36]. The enhanced expression of two ATP synthase subunits functioning in ATP synthesis and one acetate-CoA ligase (acetyl CoA synthetase) providing acetyl-CoA for biosynthesis in the transketolase II-overexpressing strain indicates a possible role for transketolase II in carbon metabolism, as distinct from transketolase I.

**Figure 6:**
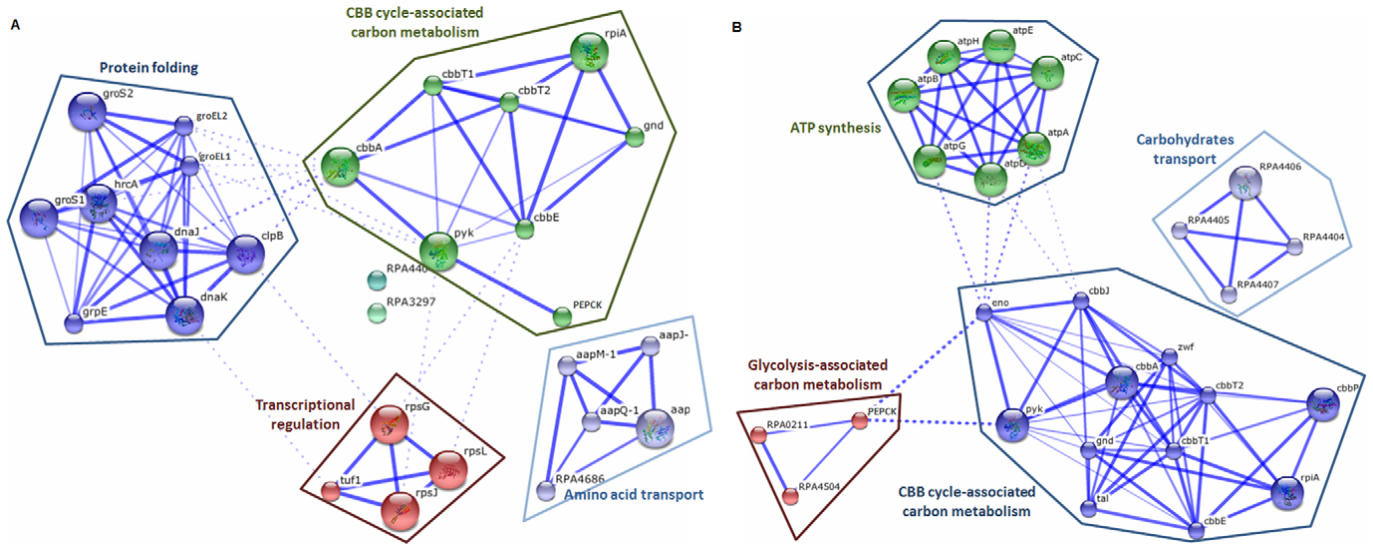
Protein-protein interaction networks (PIN) in transketolase I and II-overexpressing strains. The differentially expressed proteins in transketolase I and II-overexpressing strains and their interacting partners were used to construct the network with the STRING database. All proteins displayed in the network were further analyzed for clustering with an MCL clustering algorithm. (A) The protein interaction networks of the transketolase I-overexpressing strain. (B) The protein interaction networks of the transketolase II-overexpressing strain.

To investigate further changes to protein regulation induced by transketolase over-expression, the identified proteins and those proteins with which they interact were used to construct protein interaction networks and were analyzed for clustering to reveal key functional relationships (Figure 6). Protein folding, transcriptional regulation, amino acid transport systems and CBB cycle-associated carbon metabolism were found to be enriched in the transketolase I-overexpressing strain. In contrast, ATP synthesis, carbohydrate transport, glycolysis-associated carbon metabolism and CBB cycle-associated carbon metabolism were enriched in the transketolase II-overexpressing strain. Based on the protein profiles and protein interaction networks, the expression of proteins associated with ATP synthesis (ATP synthase subunits) and glycolysis/gluconeogenesis (acetyl-CoA synthetase, etc.) implies a tighter connection between transketolase II and glycolysis than transketolase I and glycolysis. Since transketolase-facilitated metabolism can connect the pentose phosphate pathway to glycolysis [37], we postulated that transketolase II might participate more in glycolysis than transketolase I.

To detect the changes in the rate of progression of the glycolysis cycle or ATP synthesis, we measured the ATP synthetic activity, mainly resulting from glycolysis [38], [39], in the two transketolase-overexpressing strains. In this study, we used a combination of osmotic shock and Triton X-100 (a non-inhibitory detergent used in the detection of luciferase activity) to produce permeable cells. The rate of ATP biosynthesis was calculated from the slope of the plot of luminescence against OD_660_, indicating the concentration of permeable cells. The intensity of bioluminescence at each measurement point was transformed to static ATP concentration by a standard curve fitting procedure. Our results revealed a significant increase in ATP synthetic activity in the transketolase II-overexpressing strain (ATP synthesis activity, cbbT1, 0.92+/−0.003; cbbT2, 2.06+/−0.021; NC, 0.92+/−0.0028 nM ATP/min/OD_660_), suggesting a higher rate of glycolysis. These results suggest that over-expression of transketolase II might lead to higher ATP synthesis in the cytoplasm.

According to the protein interaction networks, both transketolase I and transketolase II-overexpressing strains showed a link between CBB proteins and phosphoenolpyruvate carboxykinase (PEPCK) via pyruvate kinase (Pyk). PEPCK was highly upregulated in transketolase-overexpressing strains as shown in Figure 5D. A previous study showed that PEPCK mediated the decarboxylation of oxaloacetate (OAA) producing phosphoenolpyruvate (PEP) in gluconeogenesis or the reverse reaction in glycolysis with supplemental CO_2_ [40]. Considering the possible function of PEPCK as a CO_2_ fixing enzyme during OAA synthesis from phosphoenolpyruvate (PEP) in some algae [41]–[43], we examined whether the upregulated PEPCK possessed CO_2_-fixing activity in transketolase-overexpressing strains. We found that *in vitro*, activity of PEPCK measured in the carboxylation direction was elevated in both overexpressing strains under photoautotrophic conditions (Table 1).

**Table 1:** Activities of transketolase and PEPCK in transketolase I, transketolase II and wild type with empty vector (NC) over-expression *R. palustris* strains.

Strains	Transketolase I	Transketolase II	NC
Transketolase activity (NADH µmole/min/mg protein)	13.46±0.86	11.79±0.26	3.27±0.20
PEPCK activity (ATP nmole/min/mg protein)	129.9±5.5	110.04±6.1	65.255±3.3

Values are shown as mean+-standard deviation. All samples were harvested after 9 days of culture.

Taken together, our results suggest that overexpression of transketolase I drives the photosynthetic process, whereas overexpression of transketolase II speeds up glycolysis. These findings indicate the different roles played by the two transketolase isoforms in carbon metabolism. Transketolase I and II overexpression increased the transketolase activities and further induced the expression of PEPCK. This led to cellular redox stress alleviation and improved CO_2_ assimilation, thereby contributing to autotrophic growth in *R. palustris*.

## Discussion

In the present study, we revealed that transketolase could enhance autotrophic growth in *R. palustris*, possibly through the enhancement of CBB cycle efficiency. Current studies of the CBB cycle of photosynthetic bacteria focus on CBB cycle regulation and the main CO_2_-fixing protein, RubisCO. However, the other CBB proteins that may enhance autotrophic growth remain undefined. Our study showed that overexpression of transketolase leads to a significant biomass increase in *R. palustris*, suggesting the potential importance and functional involvement of transketolase in photoautotrophic growth. Most enzymes in the CBB cycle have been well characterized and functionally analyzed, but little is known about the structural and enzymatic characteristics of different transketolase isoforms in bacteria. In most plants, transketolase activity in the CBB cycle is mostly located in the plastid membrane where photosynthesis takes place [26]. Another study has shown that decreased transketolase activity in the plastid membrane can cause a decrease in photosynthesis, suggesting that transketolases in the CBB cycle have an effect on photosynthesis [44]. Transketolase I was preferentially located within or around the ICM, whereas transketolase II was seen mainly in the cytoplasm. The different localization of the two transketolase isoenzymes suggests they may regulate photoautotrophic growth in different ways.

Proteomic analysis combined with protein activity studies was used to reveal the proteins regulated by the two transketolase isoforms in autotrophic growth. In the protein interaction network results, CBB cycle-associated carbon metabolism was enriched in both transketolase I and II-overexpressing strains. However, protein folding, amino acid transport and transcriptional regulation were only enriched in the transketolase I-overexpressing strain. In addition, transketolase II showed a stronger connection with glycolysis and carbohydrate transport systems. These results indicate that transketolase I and II have similar functions although they employ slightly different metabolic mechanisms. Moreover, the higher involvement of glycolysis-related proteins in transketolase II-overexpressing strain observed in protein interaction network results was similar with the results of gene ontology distribution obtained from the transcriptomic study. Some functional terms related to glycolysis and carbonhydrate metabolism such as the glyceraldehyde-3-phosphate dehydrogenase (phosphorylating) activity and 4-alpha-glucanotransferase were also enriched in gene ontology analysis. The significant appearance of photosynthesis associated genes were only observed in transcriptomic analysis. It probably was resulted from the low assistance of membrane proteins, where most photosynthesis-associated molecules located, in protein extracts.

Notably, protein profiles showed that overexpression of both transketolase I and II upregulated PEPCK, a protein involved in CBB cycle-associated carbon metabolism. PEPCK is an enzyme that can reversibly catalyze oxaloacetate production from phosphoenolpyruvate via reductive carbon dioxide fixation in the bacterial cytosol [43], [45]. The higher production of oxaloacetate might lead to the enhanced synthesis of amino acids or carbohydrates [46]. Amino acid transport is an important aspect of amino acid metabolism. Several classes of permeases, members of the family of binding protein-dependent transporter systems, along with periplasmic binding proteins, are responsible for amino acid transport. In this study, we found that branched chain amino-acid ABC transporter substrate-binding protein (RPA3297) and ABC transporter, periplasmic amino acid binding protein aapJ-1 (aapJ-1) are constituents of ATP-requiring ABC transporters responsible for amino acid uptake and efflux. Amino acids such as glutamate, aspartate and histidine are all substrates for periplasmic binding protein-dependent transport systems [47], [48]. An extracellular solute-binding protein family 1 (RPA4404), was also upregulated in transketolase-overexpressing strains. RPA4404 is generally involved in the transport of nutrients specific to oligosaccharides, α-glycerol phosphate, and iron rather than amino acids [49]. Upregulation of RPA4404 suggests a higher rate of carbon metabolism that might contribute to increased autotrophic growth in *R. palustris*.

In the transketolase II-overexpressing strain, ATP synthases and acetate-CoA ligase were upregulated. Acetate-CoA ligase is an important enzyme involved in the glycoxylic acid cycle that can catalyze the conversion of acetate into acetyl-coenzyme A (acetyl-coA). The acetyl-CoA can be metabolized for the synthesis of succinate and malate via the glycoxylic cycle [50]–[52], which might be an alternative means of facilitating autotrophic growth ability. The upregulation of these proteins in the transketolase II-overexpressing strain resulted in an increase in ATP synthesis.

Two chaperonins (GroEL and DnaK) were up-regulated in the transketolase I-overexpressing strain. Chaperonins are specialized proteins that assist in carrying structural information from DNA to form biologically active proteins. GroEL is highly conserved in a variety organisms and is necessary for preventing protein misfolding and erratic multi-molecular aggregation [53]–[56], and is especially important for the normal folding of large protein complexes, such as RubisCO [53]. *In vitro* studies have shown that GroEL facilitates the reconstitution of the native form of RubisCO in the purple non-sulfur bacteria *R. sparoids* and *R. rubrum* [57]–[59], suggesting that GroEL might be a regulator of the CBB cycle and of autotrophic growth [60], [61].

In conclusion, our findings show that the two isoforms of transketolase in *R. palustris* had an influence on autotrophic growth via two different mechanisms reflecting on their functional differences. Transketolase I may participate in photosynthesis to generate energy for the CBB cycle. In contrast, transketolase II may be mainly responsible for the pentose phosphate pathway, which promotes carbon flow to the glycolysis pathway and provides an energy supply for the CBB cycle. Moreover, transketolase I and II may be the regulators of oxaloacetate synthesis, catalyzed by PEPCK. In this study, we shed light on some of the functional roles of the two transketolase isoforms, I and II, enhancing CBB cycle efficiency and inducing PEPCK involvement, leading to the enhancement of photoautotrophic growth in *R. palustris*.

## Materials and Methods

### Bacterial strains and culture conditions

*E. coli* TOP10 cells were used for cloning and for protein expression analysis. The conjugative strain *E. coli* S17 was employed for transforming constructs in *R. palustris*. The bacterial strains used in this study are listed in Table S1. *E. coli* cultures were grown aerobically at 37°C in Luria-Bertani (LB) broth and supplemented with antibiotics when required. *R. palustris* CGA010 was derived from GCA009 and consisted of a repaired frameshift mutation in the *hupV* gene. For photolithoautotrophic growth, the *R. palustris* strain was grown anaerobically in light. Cells were cultured in PF-7 medium containing ammonium sulfate as described previously [62] and Na_2_HCO_3_ (Sigma-Aldrich Corp., St. Louis, MO, USA) was used as the sole carbon source; cells were maintained at 30°C in 100 mL crimp-sealed bottles with 95% N_2_ and 5% CO_2_ headspace. Photoheterotrophic growth was carried out in 20 mL crimp-sealed tubes containing photomixotrophic medium (PM) [63] modified by the addition of 10 mM succinate (J.T Baker, Phillipsburg, NJ), ammonium sulfate (Sigma-Aldrich), 0.2% yeast extract (Laboratorios Conda, S.A., Madrid, Spain) and 0.5% casamino acid (Becton, Dickinson and Company, Sparks, MD, USA) under anaerobic light conditions. All anaerobic light cultures were illuminated with 60 W–70 W incandescent lamps from multiple directions with a light intensity of 34∼35 W/m^2^. Antibiotics were used at the following concentrations: 40–50 mg/mL gentamycin (Bio Basic Inc., Ontario, Canada) and 30 mg/mL chromophenicol (Bioshop Canada Inc., Ontario, Canada). LB and PM solid media contained 1.5% agar (wt/vol).

### Plasmid construction and DNA manipulation

Plasmids generated in this study are listed in Table S1. A gentamycin-resistant plasmid pBBR1MCS-5 [28] was used for all constructions. Plasmid DNA purification, PCR, restriction digestion and cloning were performed according to the manufacturer's protocols. Plasmid DNA was purified using a Bioman plasmid purification kit (Bioman Scientific Co., Ltd., Taipei, Taiwan) according to the manufacturer's instructions. Phusion High-Fidelity DNA Polymerase (Finnzymes, Espoo, Finland) was used for amplifying genomic DNA. Restriction enzymes were obtained from New England Biolabs (Beverly, MA, USA) and Fermentas (Vilnius, Lithuania). T4 DNA ligase was purchased from RBC Bioscience (Chung Ho City, Taiwan). Primers with appropriate restriction sites were used for PCR amplification of regions flanking the genes of interest. The primer sequences will be provided upon request. The native promoters or the *pucBe* promoter containing a ribosomal-binding site were cloned into a pBBRMCS-5 plasmid for cbb gene transcription. The amplified products containing engineered XbaI and XhoI or ApaLI and XbaI cloning sites were digested with XbaI and XhoI/ApaLI and cloned into XbaI/ApaLI-digested pBBR1MCS-5 to generate pMCS-5-cbbLS andpMCS-5-cbbT2, or XbaI/XhoI-digested pBBR1MCS-5 with *pucBe* promoter to generate pMCS-5-cbbP, pMCS-5-cbbA, pMCS-5-cbbT1 and pMCS-5-cbbF. The constructs were transformed into the conjugative strain S-17 and introduced into *R. palustris* [14], [64]. Exoconjugants harboring a chromosomal insertion of the plasmid were selected for chromophenicol and gentamycine resistance to confirm recombination. For immunogold electron microscopy, transketolase was fused to nine amino acids hemagglutinin (HA) tag on the N-terminal. All the plasmids generated in this study were confirmed by PCR and sequencing. Protein over-expression for each strain was confirmed by sodium dodecyl sulfate polyacrylamide gel electrophoresis (SDS-PAGE) on a 10% polyacrylamide gel.

### Dry cell weight determination and growth curves

This analysis was performed based on the procedure previously described, with some minor modifications [31], [65]. The initial cell number for each bacterial strain was normalized, as determined by absorbance at 660 nm. The bacteria were cultured under photoautotrophic conditions for 9 days then the dry cell weight was determined by filtering the known volumes of bacterial cultures through pre-weighed 0.22 µm cellulose nitrate membrane filters. All membrane filters were dried to achieve a final constant weight before use. After filtering, each membrane was again dried to a constant weight at 65°C, and the final dry weight was recorded. The measurement of growth curves was performed in 25 mL crimp-sealed tubes with a 95% N_2_ and 5% CO_2_ headspace. The bacteria were grown in PF-7 medium with ammonium sulfate and incubated at 30°C. The OD_660_ of each tube was monitored using spectrophotometry. The growth curve of the CBB gene-overexpressing strains was compared to a negative control strain with an empty vector grown under photoautotrophic conditions.

### Absorption spectrum

The bacteria, cultured under photoautotrophic conditions, were collected and washed in a low-salt phosphate buffered saline (PBS). The pellets were resuspended in 0.05% DDM solution (0.05% dodecyl maltoside, 50 mM Tris-HCl, 500 mM NaCl) and lyzed by sonication. The cell debris was removed by centrifugation and the concentration of the extracted protein was measured with Bradford's method using a protein assay kit (Bio-Rad, Hercules, CA, USA). The absorbance from 300 nm to 900 nm was measured in a total of 600 µg of protein.

### Real-time quantitative PCR

*R. palustris* was grown in photoautotrophic conditions until the OD_660_ reached 1.5. The bacteria were collected and homogenized by sonication and the total RNA was extracted with an RNA mini kit (QIAGEN, Hilden, Germany) according to the manufacturer's instructions. RNA was quantified using a NanoDrop® ND-1000 Spectrophotometer (Thermo Fisher Scientific, Wilmington, DE, USA). Reverse transcription was performed using a ThermoScript™ RT-PCR Systems Kit (Invitrogen Corporation, Carlsbad, CA, USA) according to the manufacturer's instructions. The reaction mixture was subjected to subsequent incubation steps: 5 min at 65°C, 50 min at 55°C, 5 min at 85°C and an additional 20 min at 37°C after adding RNaseH. Quantitative PCR was performed using iQ SYBR Green Supermix (Bio-Rad). Assays were performed in a 20 µL final volume with 10 µL of SYBR Green Supermix, 0.4 µL of each primer and 4 µL of 1000-fold-diluted cDNA or water as a negative control. Thermal cycling was initiated at 95°C for 3 min and followed by 10 sec at 95°C, 30 sec at 60°C and 1 min at 95°C. The sequences of the primers used in this analysis are listed in Table S5. Gene expression of each strain, cbbT1 and cbbT2, was first normalized by relative quantification using the control transcripts of *rpoD* [66] and then divided by the expression value of negative control to obtain a relative gene expression ratio.

### Immunogold electron microscopy

For immunogold labeling, the two strains containing the fusion proteins cbbT1-HA and cbbT2-HA were used for protein localization. The expression of the HA-tagged recombinant protein was confirmed by Western blot. Cells grown under photoautotrophic conditions were dropped onto a copper (Cu) grid and stained with 1% phosphotungstic acid (PTA) for negative staining. The grids were dried and visualized using Topographic Electron Microscopy (TEM) (HITACH H-7650). For London Resin (LR) White embedding, the grids were fixed, washed and dehydrated in solutions of ethanol of increasing concentrations up to 100%. The embedding process was performed using medium-grade resin and pure LR white, followed by further embedding in gelatin capsules and polymerization at 55°C for 2 days. The ultrathin section and immunogold labeling methods were performed according to previously described methods with some modification [67], [68]. During the ultrathin sectioning process, sections with a thickness of 85 nm were cut and mounted on nickel or copper grids (100 or 150 mesh with a Formvar membrane). For immunogold labeling, the grids were immersed in a PBS solution with 20 mM glycine for 30 min to block non-specific labeling and then incubated with primary anti-HA antibody (Sigma) for 1 hr (at 1∶20 or 1∶40 dilutions). The secondary antibody was conjugated with 10 nm gold particles and incubated for 1 hr.

The ultrathin sections were stained with a 2% solution of uranyl acetate with lead citrate to increase the contrast and were observed with transmission electron microscopy (TEM). For quantitation of ICM abundance, a total of 326 longitudinal-section micrographs (magnificantion 50000×) from 5–6 grids were counted for the percentage frequency of bacteria with obvious ICM structure observed in each strain. Quantification of immunogold labeling in distinct cellular parts was performed on thirty different bacterial sections (15 longitudinal and 15 cross-sectional) of each strain. Each section was evaluated with TEM to determine the extent of the ICM associated regions. A negative control was also visualized using the same procedure.

### Protein Extraction

*R. palustris* cells were collected by centrifugation and the cell pellets were washed twice with low-salt PBS (3 mM KCl, 1.5 mM KH_2_PO4, 68 mM NaCl, 9 mM NaH2PO4). Cells were resuspended in 500 µL of lysis buffer containing 7 M urea (Ameresco, Solon, OH, USA), 2 M thiourea (Boehringer, Mannheim, Germany), 4% CHAPS (J.T Baker), 5 µL protease inhibitor cocktail (Bioman Scientific Co., Ltd) and then lyzed by sonication. The concentration of the extracted protein was measured by Bradford's method using a protein assay kit (Bio-Rad).

### Two-dimensional electrophoresis (2DE)

Prior to electrophoresis, total proteins (350 µg) were mixed with rehydration buffer containing 7 M urea (Ameresco), 2 M thiourea (Boehringer), 4% CHAPS (J.T Baker), 65 mM DTE (AppliChem, Darmstadt, Germany), 0.5% bromophenol blue (Ameresco) and pH 3–10 NL IPG Buffer (Bio-Rad) to a total volume of 315 µL. The first dimension, or isoelectric focusing (IEF) step, was performed using an Ettan IPGphor II system (Amersham Pharmacia Biotech, Uppsala, Sweden) as previously described [69]. The protein mixture was loaded onto an 18 cm pH 4–7 gradient immobiline DryStrip (Bio-Rad) and the rehydration step was carried out for 12 h at 50 µA/strip at 20°C. IEF was performed using the following phases: (1) 100 V for 1 h; (2) 250 V for 1 h; (3) 500 V for 1 h; (4) 1000 V for 1 h; (5) 4000 V for 1 h; and (6) 8000 V for a total of 65 kVh. After reduction with 65 mM DTE and alkylation with 55 mM iodoacetamide, the strips were transferred to 12–18% gradient acrylamide gels (Bioshop Canada Inc.). The second dimensional separation was performed using a Protean II XL (Bio-Rad) apparatus with the current set at 40 mA per gel. The protein gel was fixed in 10% methanol and 7% acetic acid and stained with SYPRO Ruby (Invitrogen Corporation, Carlsbad, CA, USA). Images of the stained gels were made with a Typhoon 9200 Fluorescence Imager (Amersham Pharmacia Biotech) and analyzed using the Image Master 2D elite software package (Amersham Pharmacia Biotech) in high image quality TIF format.

### In-gel digestion

For gel-to-gel comparison, the 2D image of the wild type *R. palustris* was used as the reference gel image. Quantitative comparison of protein spots based on their percent volumes was performed after spot-matching between cells transformed with the overexpression vectors and cells transformed with the control vector. The expression ratios of protein spots were determined using the volumes of the image spots. For in-gel digestion, protein spots of interest were manually excised and washed with 1∶1 (v/v) solution containing 50 mM ammonium bicarbonate and acetonitrile (ACN). After treatment with Na_2_CO_3_, proteins were digested with sequence-grade trypsin (Promega Corporation, Madison, WI, USA) for 16 hr at 37°C. For peptide extraction after in-gel digestion, a solution containing 1% trifluoroacetic acid (TFA) in 50% ACN was added and the combined extracts were allowed to dry. The peptides were eluted with 0.1% TFA in 2% ACN and deposited onto the MALDI plate (PerSeptive Biosystems, CA, USA) of a MALDI-TOF mass spectrometer.

### Protein identification

Matrix-assisted laser desorption/ionization-time-of-flight mass spectrometry (MALDI-TOF MS) or MS/MS was performed on a dedicated Q-Tof Ultima MALDI instrument (Micromass, Manchester, UK) with fully automated data-directed acquisition using a predefined probe motion pattern and peak intensity threshold for switching over from MS survey scanning to MS/MS, and from one MS/MS to another.

The peak list was acquired using MassLynx™ software version 4.0 and the raw data were processed using ProteinLynx Global Server 2.2 (PLGS2.2) to enable database searches. MS/MS data were collected from every sample. Within each sample well, parent ions that met the predefined criteria (any peak within the *m*/*z* range of 80–3000 and with an intensity of above 10 counts ± include/exclude list) were selected for CID MS/MS using argon as the collision gas and a mass dependent±5 V rolling collision energy until the end of the probe pattern was reached (all details are available at http://proteome.sinica.edu.tw). The output of each individual MS/MS data point from every sample well was represented as a single MASCOT-searchable peak list. The LM and HM resolutions of the quadrupole were both set at 10 to give a precursor selection window of approximately 4 Da. The instrument was calibrated to less than 5 ppm accuracy over the mass range of m/z 800–3000 using sodium iodine and PEG 200, 600, 1000 and 2000 mixtures and was further adjusted with Glu-Fibrinopeptide B as the near-point kick mass calibrant during data processing. At a laser firing rate of 10 Hz, individual spectra from a 5 second integration period, which was acquired for each of the MS surveys, and MS/MS results were combined, smoothed, deisotoped (fast option) and centroided using the Micromass PKG 2.2 data processing software. The combined peptide mass fingerprinting (PMF) and MS/MS meta data were searched for protein identification. Subsequently, proteins were identified by searching in the NCBI database using the MASCOT (http://www.matrixscience.com) search engine (peptide mass fingerprint and MS/MS ion search). The search parameters were set as follows: peptide mass tolerance was 50 ppm; fragment mass tolerance was 0.25 Da; only tryptic peptides with up to one missed cleavage site were allowed; modifications were carbamidomethylation (C) and oxidation of methionine. For positive identification, a given result [−10 Log (*P*)] had to be over the significance threshold level (*P<0.05*).

### Construction of functional interaction networks

Gene symbols of over-represented signaling pathways were loaded into the database STRING for construction of functional interaction networks [70], [71]. Reported interactions contain direct (physical) and indirect interactions based on experimental evidence, co-regulated gene expression, the same genomic context or co-citation in the literature. Only interactions with a minimum STRING score of 0.400, the default medium confidence level in STRING, were used in this study. To cluster the proteins displayed in the networks, an MCL clustering algorithm was utilized to launch the clustering [72], [73].

### Cellular ATP synthetic activity analysis

Cellular ATP synthesis was analyzed as previously described [38]. Cells grown under photoautotrophic conditions were washed and resuspended in 100 mM Tris-HCl (pH 7.4) with the OD_660_ adjusted to 0.4. The cell suspension was mixed with an equal volume of pretreatment solution (40% [w/v] glucose, 0.8% [v/v] Triton X-100) for 20 min at room temperature (Mixture C).

The ATP assay solution (SIGMA), which was pre-incubated for 15 min at room temperature, was diluted to 0.05 of its original concentration with detergent solution (8% Triton-100, 300 mM potassium phosphate [pH 7.2]); this was given the designation ‘Mixture D’. The reaction was initiated by the addition of 10 µL Mixture C to 90 µL Mixture D in a white 96-well microplate. Kinetic luminescence data were continuously measured by a SpectraMax M5 reader (Molecular Devices, Sunnyvale, CA, USA). The static ATP concentration (nM ATP/min/OD_660_) was given by L_o_/αd and the ATP synthesis activity (nM ATP/min/OD_660_) by Δ/αd. Abbreviations are as follows: L_o_, intercept of luminescence (relative luminescence units [RLU]); Δ, velocity of increasing luminescence (RLU/min); α, conversion value of luminescence (RLU/nM ATP); d, OD_660_ of cell suspensions.

### Transketolase activity assays

The activity of transketolase was calculated by monitoring the production of sedoheptulose 7-phosphate using the coupling method as previously described [37]. The activity was measured in a solution composed of 10 mM Tris-HCl (pH 7.4), 5 mM MgCl_2_, 1 mM ribose 5-phosphate, 1 mM xylulose 5-phosphate, 2 units of glycerol 3-phosphate dehydrogenase, 2 units of triose-phosphate isomerase and 0.1 mM NADH at 30°C.

### PEPCK activity assay

PEPCK activity was determined by measuring ATP formation at 30°C using the Sigma Diagnostics ATP kit (Sigma) following the manufacturer's instructions [74]. The reaction mixture (200 µl) for the PEPCK assay contained 100 mM Tris-HCl (pH 7.8), 5 mM PEP, 35 mM NaHCO_3_, 16 mM MgCl_2_, 10 mM ADP and an ATP assay premix containing luciferase and luciferin. The reaction was initiated by adding 100 µl of the cell extract and incubating the mixture at room temperature for 3 min. ATP formation was measured using a SpectraMax M5 reader (Molecular Devices). The PEPCK activity was expressed as amount of ATP (mmol) produced per mg protein per min.

## Supporting Information

Figure S1 **The effects of overexpression of different CBB proteins on the biomass production and growth curve of** ***R. palustris*** **.** A. Biomass analysis (DCW) was performed to characterize the photoautotrophic growth ability of the different strains. The initial cell number of each strain was 10^9^. Each bar represents the mean of three assays. Cultures were grown under identical conditions. NC indicates negative control strain with empty plasmid. dpc, days post culture. * *p*<0.05; ** *p*<0.005. B. The growth curves of the CBB gene-overexpressing strains. Overexpressed CBB genes included transketolase I (cbbT1), transketolase II (cbbT2), phosphoribulokinase (cbbP), fructose-1,6-bisphosphate aldolase (cbbA), ribulose 1,5-bisphosphate carboxylase/oxygenase (cbbLS) and D-fructose 1,6-bisphosphatase (cbbF). NC indicates negative control strain with empty plasmid; WT, wild type without MCS-5 plasmid.
(TIF)

Figure S2 **Electron micrographs of photoautotrophic** ***R. palustris*** **cells.** A. Left, longitudinal section of a cell; right, the resolved figures of large stacks of ICMs, cell membrane (CM), and the cell wall (CW). B, ultra-section election micrographs of negative control strain grown in photoheterotrophic condition. C.S., cross section; L.S., longitudinal section.
(TIF)

Table S1 **Bacterial strains and plasmids used in this study.**
(DOC)

Table S2 **The gene ontology distribution of differentially expressed genes in the transketolase I-overexpressing strain of** ***R. palustris*** **.** The annotations were categorized in accordance with the description of the European Bioinformatics Institute's GO Annotation database with Gossip Fisher's exact test *p*-value<0.01.
(DOC)

Table S3 **The gene ontology distribution of differentially expressed genes in transketolase II-overexpressing strain of** ***R. palustris*** **.** The annotations were categorized in accordance with the description of the European Bioinformatics Institute's GO Annotation database with Gossip Fisher's exact test *p*-value<0.01.
(DOC)

Table S4 **Identification of significantly differentially expressed proteins in transketolase I, transketolase II over-expression and wild type with empty vector (NC) R. palustris strains.**
(DOC)

Table S5 **Primers for qPCR of photosynthetic genes.**
(DOC)

Materials and Methods S1 (DOC)
