# Supplementary figures and images for: Revealing the Functions of the Transketolase Enzyme Isoforms in Rhodopseudomonas palustris Using a Systems Biology Approach
Source: PLoS One. 2011 Dec 8;6(12):e28329. doi: 10.1371/journal.pone.0028329 (PMC3234253; doi:10.1371/journal.pone.0028329)

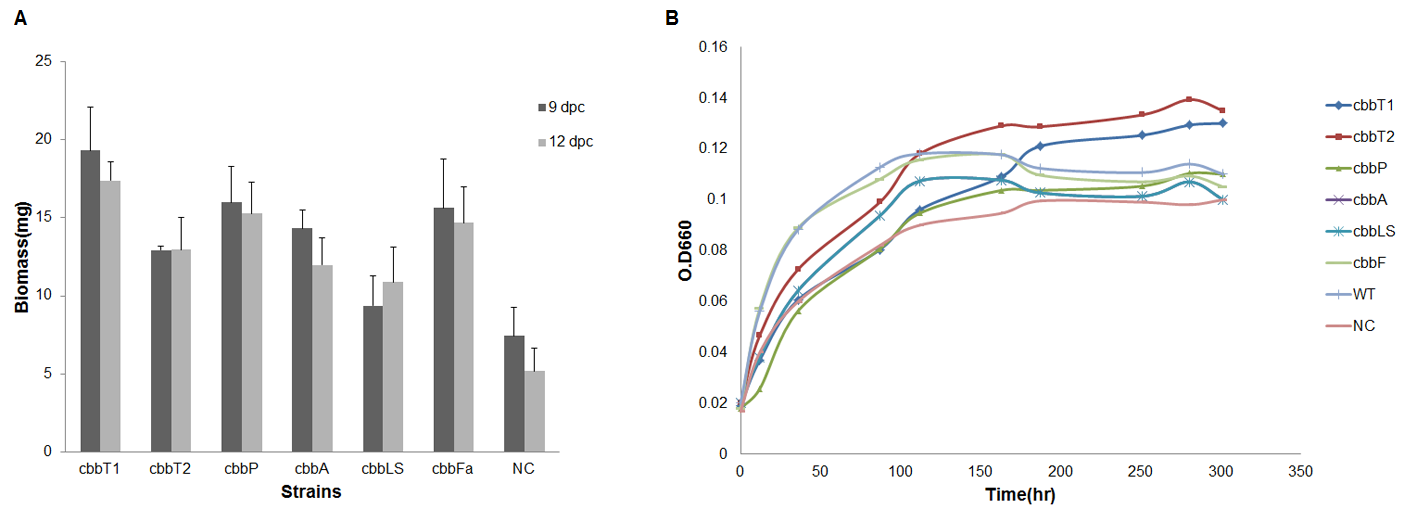

Supplement: Figure S1 — The effects of overexpression of different CBB proteins on the biomass production and growth curve of R. palustris . A. Biomass analysis (DCW) was performed to characterize the photoautotrophic growth ability of the different strains. The initial cell number of each strain was 109. Each bar represents the mean of three assays. Cultures were grown under identical conditions. NC indicates negative control strain with empty plasmid. dpc, days post culture. * p<0.05; ** p<0.005. B. The growth curves of the CBB gene-overexpressing strains. Overexpressed CBB genes included transketolase I (cbbT1), transketolase II (cbbT2), phosphoribulokinase (cbbP), fructose-1,6-bisphosphate aldolase (cbbA), ribulose 1,5-bisphosphate carboxylase/oxygenase (cbbLS) and D-fructose 1,6-bisphosphatase (cbbF). NC indicates negative control strain with empty plasmid; WT, wild type without MCS-5 plasmid. (TIF) [file pone.0028329.s001.tif]

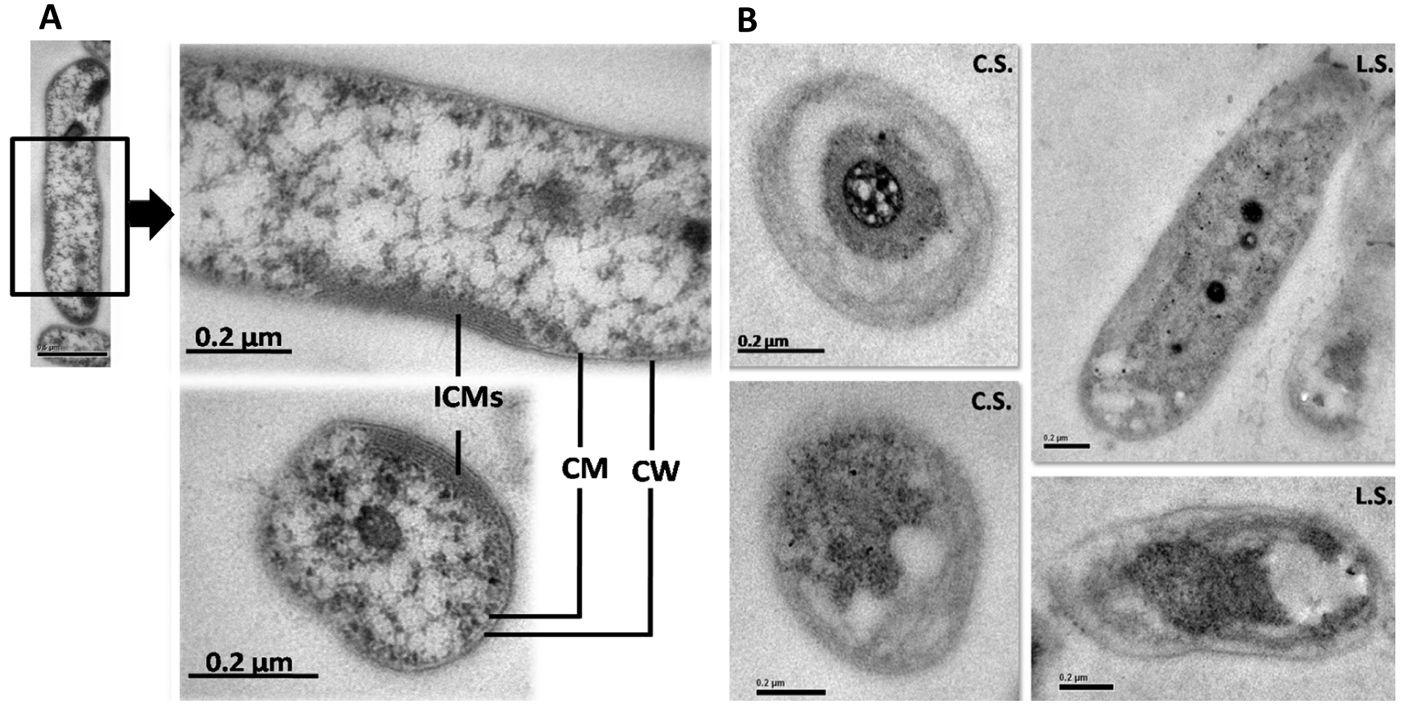

Supplement: Figure S2 — Electron micrographs of photoautotrophic R. palustris cells. A. Left, longitudinal section of a cell; right, the resolved figures of large stacks of ICMs, cell membrane (CM), and the cell wall (CW). B, ultra-section election micrographs of negative control strain grown in photoheterotrophic condition. C.S., cross section; L.S., longitudinal section. (TIF) [file pone.0028329.s002.tif]
