# Supplementary material for: Revealing the Functions of the Transketolase Enzyme Isoforms in Rhodopseudomonas palustris Using a Systems Biology Approach
Source: PLoS One. 2011 Dec 8;6(12):e28329. doi: 10.1371/journal.pone.0028329 (PMC3234253; doi:10.1371/journal.pone.0028329)
Supplement: Table S1 — Bacterial strains and plasmids used in this study. (DOC) [file pone.0028329.s003.doc]

**Table S1.** Bacterial strains and plasmids used in this study.

| **Strains** | **Relevant characteristics** | **Source or reference** |
| --- | --- | --- |
| *E. coli* |  |  |
| TOP10 | Cloning strain and plasmid and replication host | Invitrogen |
| S17 | Conjugative host | S3 |
| *R. palustris* |  |  |
| CGA010 | Wild type | S3 |
| cbbT1 | Transketolase I *(cbbT1)*-overexpressing strain | This study |
| cbbT2 | Transketolase II *(cbbT2)*-overexpressing strain | This study |
| cbbP | Phosphoribulokinase *(cbbP)*-overexpressing strain | This study |
| cbbA | Fructose-1,6-bisphosphate aldolase *(cbbA)*-overexpressing strain | This study |
| cbbLS | Bisphosphate carboxylase/oxygenase *(cbbLS)*-overexpressing strain | This study |
| cbbF | D-fructose 1,6-bisphosphatase *(cbbF)*-overexpressing strain | This study |
| NC | Wild type with empty plasmid | This study |
| *Plasmids* |  |  |
| pJRB741 | pBBR MCS-5, control bacteria | S4 |
| pJRB735 | pBBR MCS-5 with *cbbT1* | This study |
| pJRB706 | pBBR MCS-5 with *cbbT2* | This study |
| pJRB737 | pBBR MCS-5 with *cbbP* | This study |
| pJRB733 | pBBR MCS-5 with *cbbA* | This study |
| pJRB701 | pBBR MCS-5 with *cbbLS* | This study |
| pJRB734 | pBBR MCS-5 with *cbbF* | This study |
