# Supplementary material for: Revealing the Functions of the Transketolase Enzyme Isoforms in Rhodopseudomonas palustris Using a Systems Biology Approach
Source: PLoS One. 2011 Dec 8;6(12):e28329. doi: 10.1371/journal.pone.0028329 (PMC3234253; doi:10.1371/journal.pone.0028329)
Supplement: Table S2 — The gene ontology distribution of differentially expressed genes in the transketolase I-overexpressing strain of R. palustris . The annotations were categorized in accordance with the description of the European Bioinformatics Institute's GO Annotation database with Gossip Fisher's exact test p-value<0.01. (DOC) [file pone.0028329.s004.doc]

**Table S2. Gene ontology distribution of differentially expressed genes in the transketolase I-overexpressing strain of *R. palustris*.** The annotations were categorized in accordance with the description of the European Bioinformatics Institute’s GO Annotation database with Gossip Fisher’s exact test *p*-value < 0.01.

| **Functional annotation*** | ***p*-Value** |
| --- | --- |
| Plasma membrane-derived chromatophore membrane | 1.08E-04 |
| Photosynthetic membrane | 1.08E-04 |
| Monooxygenase activity | 4.85E-04 |
| Plasma membrane light-harvesting complex | 9.30E-04 |
| Cytoplasmic vesicle part | 9.30E-04 |
| Electron transporter, transferring electrons within the cyclic electron transport pathway of photosynthesis activity | 9.30E-04 |
| Light-harvesting complex | 9.30E-04 |
| Photosynthetic electron transport in photosystem II | 0.001058 |
| Photosynthetic electron transport chain | 0.001058 |
| Bacteriochlorophyll biosynthetic process | 0.001086 |
| Bacteriochlorophyll metabolic process | 0.001086 |
| Oxidation reduction | 0.001387 |
| Protochlorophyllide reductase activity | 0.002193 |
| Oxidoreductase activity, acting on the CH-CH group of donors, NAD or NADP as acceptor | 0.002372 |
| Oxidoreductase activity | 0.003106 |
| Chlorophyll biosynthetic process | 0.003598 |
| Chlorophyll metabolic process | 0.003598 |
| Oxidoreductase activity, acting on paired donors, with incorporation or reduction of molecular oxygen, NADH or NADPH as one donor, and incorporation of one atom of oxygen | 0.00371 |
| Heterocycle biosynthetic process | 0.003757 |
| Cofactor metabolic process | 0.003773 |
| Cofactor biosynthetic process | 0.005049 |
| Organelle membrane | 0.006668 |
| Organelle inner membrane | 0.006668 |
| Porphyrin biosynthetic process | 0.008175 |
| Porphyrin metabolic process | 0.008941 |
