# Supplementary material for: Revealing the Functions of the Transketolase Enzyme Isoforms in Rhodopseudomonas palustris Using a Systems Biology Approach
Source: PLoS One. 2011 Dec 8;6(12):e28329. doi: 10.1371/journal.pone.0028329 (PMC3234253; doi:10.1371/journal.pone.0028329)
Supplement: Table S3 — The gene ontology distribution of differentially expressed genes in transketolase II-overexpressing strain of R. palustris . The annotations were categorized in accordance with the description of the European Bioinformatics Institute's GO Annotation database with Gossip Fisher's exact test p-value<0.01. (DOC) [file pone.0028329.s005.doc]

**Table S3. Gene ontology distribution of differentially expressed genes in the transketolase II-overexpressing strain of *R. palustris.*** The annotations were categorized in accordance with the description of the European Bioinformatics Institute’s GO Annotation database with Gossip Fisher’s exact test *p*-value < 0.01.

| **Functional annotation*** | ***p*-Value** |
| --- | --- |
| transferase activity, transferring hexosyl groups | 0.001919 |
| DNA packaging | 0.002429 |
| 4-alpha-glucanotransferase activity | 0.002429 |
| glyceraldehyde-3-phosphate dehydrogenase (phosphorylating) activity | 0.004853 |
| transketolase activity | 0.004853 |
| glyceraldehyde-3-phosphate dehydrogenase activity | 0.004853 |
| 4-hydroxyphenylpyruvate dioxygenase activity | 0.004853 |
| glycogen debranching enzyme activity | 0.007272 |
| 1,4-alpha-glucan branching enzyme activity | 0.007272 |
| transferase activity, transferring glycosyl groups | 0.008912 |
