# Supplementary material for: Revealing the Functions of the Transketolase Enzyme Isoforms in Rhodopseudomonas palustris Using a Systems Biology Approach
Source: PLoS One. 2011 Dec 8;6(12):e28329. doi: 10.1371/journal.pone.0028329 (PMC3234253; doi:10.1371/journal.pone.0028329)
Supplement: Table S4 — Identification of significantly differentially expressed proteins in transketolase I, transketolase II over-expression and wild type with empty vector (NC) R. palustris strains. (DOC) [file pone.0028329.s006.doc]

**Table S4. Identification of significantly differentially expressed proteins in transketolase I, transketolase II over-expression and wild type with empty vector (NC) *R. palustris*** strains.

| **Spot** | **Gene name** | **Accession number** | **Protein name** | **Theo. MW/PI** | | **Score** | **Matched peptides** | **Sequence**  **Coverage (%)** | **Peptides identified** | **Fold change**  **(mean±SD)** | **Molecular function** |
| --- | --- | --- | --- | --- | --- | --- | --- | --- | --- | --- | --- |
| **Up-regulated proteins in transketolase I over-expression strain** | | | | | | | | | | | |
| 1 | *atpD* | gi39933253 | F0F1 ATP synthase subunit beta | | 50799/5.07 | 244 | 3 | 10 | GQEVTDTGAPISVPVGAGTLGR  TVLIQELINNVAR  DQGQDVLFFVDNIFR | 2.12 ± 0.084 | ATP synthesis |
| 2 | *groEL* | gi39935233 | Chaperonin GroEL | | 57759/ 5.38 | 220 | 4 | 9 | SADAAGDGTTTATVLAQAIVR  SLETELDVVEGMQFDR+Oxidation  MRVEFDDAYILINEK +Oxidation  AAVEEGIVPGGGVALLR | 1.6± 0.035 | Protein folding |
| 3 | *dnaK* | gi39933410 | Chaperone DnaK | | 68019/5.11 | 246 | 3 | 8 | VDQAVITVPAYFNDAQR  DAGLTAGEISEVVLVGGMTR  MLGQFDLMGIPPAPR | 1.94± 0.013 | Protein folding |
| 5 | *pckA* | gi39933437 | Phosphoenolpyruvate carboxykinase | | 59077/5.9 | 200 | 5 | 14 | NGEAVLSSDGALVADTGVFTGR  AELENFVPELTLIDLPSFR  SENVVAIDFAR  SAYPLESIPNASLTGR  ALLTAALDGSLR | 1.61± 0.035 | Gluconeo- genesis |
| 7 | *RPA 4404* | gi39937464 | Extracellular solute-binding protein, family 1 | | 66785/7.06 | 57 | 2 | 6 | SHVGLTFIR  LGGLIEFYR | 1.37± 0.014 | Transport |
| 8 | *tufA* | gi39936315 | Elongation factor Tu | | 43309/5.53 | 200 | 3 | 8 | VGDEIEIVGIRDTQK  KLLDQGQAGDNIGALLR  LLDQGQAGDNIGALLR | 1.63± 0.06 | Protein synthesis |
| 9 | *cbbT1* | gi39937701 | Transketolase | | 68864/6.15 | 67 | 2 | 4 | LAGKLSADYEG  AKKSDRPSL | 1.49± 0.057 | Carbon metabolism |
| 10 | *RPA 3297* | gi39936360 | Branched chain amino-acid ABC transporter substrate-binding protein | | 48275/7.57 | 42 | 2 | 5 | VIAALEGFEFDGLGNGKT  LEGFEFDGLGN | 1.49± 0.014 | Amino acid transport |
| 12 | *aapJ-1* | gi39935626 | ABC transporter, periplasmic amino acid binding protein aapJ-1 | | 36730/6.22 | 46 | 2 | 4 | DRFTALQSGEIDVLSR  QSGEIDVLSRNTT | 1.87± 0.41 | Transport |

**Table 1.** Continued

| **Spot** | **Gene name** | **Accession number** | | **Protein name** | **Theo. MW/PI** | | **Score** | **Matched peptides** | **Sequence**  **Coverage (%)** | **Peptides identified** | **Fold change**  **(mean±SD)** | **Molecular function** |
| --- | --- | --- | --- | --- | --- | --- | --- | --- | --- | --- | --- | --- |
| **Up-regulated proteins in transketolase II over-expression strain** | | | | | | | | | | | | |
| 1 | *atpD* | gi39933253 | F0F1 ATP synthase subunit beta | | 50799/5.07 | | 244 | 3 | 10 | GQEVTDTGAPISVPVGAGTLGR  TVLIQELINNVAR  DQGQDVLFFVDNIFR | 2.15± 0.85 | ATP synthesis |
| 4 | *RPA 0211* | gi39933288 | Acetate--CoA ligase | | | 73040/5.77 | 95 | 4 | 4 | IGAIHSVVFAGFSPDSLAGR  VNIFYTAPTAIR SAYPLESIPNASLTGR  ALLTAALDGSLR | 1.59±0.014 | Metabolic process |
| 5 | *pckA* | gi39933437 | Phosphoenolpyruvate carboxykinase | | | 59077/5.9 | 200 | 5 | 14 | NGEAVLSSDGALVADTGVFTGR  AELENFVPELTLIDLPSFR  SENVVAIDFAR  SAYPLESIPNASLTGR  ALLTAALDGSLR | 1.61± 0.64 | Gluconeo- genesis |
| 6 | *atpA* | gi39933255 | F0F1 ATP synthase subunit alpha | | | 55314/6.14 | 130 | 3 | 9 | NFGQEAEVSEVGQVLSVGDGIAR  EAYPGDVFYLHSR  AFEDGLLALLR | 1.49± 0.57 | ATP synthesis |
| 7 | *RPA 4404* | gi39937464 | Extracellular solute-binding protein, family 1 | | | 66785/7.06 | 57 | 2 | 3 | SHVGLTFIR  LGGLIEFYR | 1.44±0.077 | Transport |
| 11 | *cbbT2* | gi39934022 | Transketolase | | | 70382/5.81 | 67 | 2 | 6 | LAGKLSADYEG  AKKSDRPSL | 1.72±0.084 | Carbon metabolism |
| **Down-regulation in transketolase I and transketolase II over-expression strains** | | | | | | | | | | | | |
| 13 | *RPA 1811* | gi39934880 | Enoyl-CoA hydratase/isomerase | | 27773/5.35 | | 41 | 2 | 11 | STFEFIIVER  FGQPEITLGTIPGIGGTQR | 0.39±0.12 (cbbT2/NC)  0.35±0.23  (cbbT2/NC) | Metabolic process |
| 14 | *RPA 3070* | gi39649987 | Conserved unknown protein | | 32195/5.32 | | 58 | 2 | 8 | SIDLELTNALAMK AADKGSSAAMVELGVAYATGVGLPK | 0.28±0.085  (cbbT1/NC)  0.19±0.073  (cbbT2/NC) | Metabolic process |
| 15 | *RPA 2604* | gi39935670 | Peptidyl-prolyl cis-trans isomerase, cyclophilin type | | 16916/5.63 | | 97 | 2 | 23 | EGFYDGIVFHR  LKAEFNAEPHVR | 0.32±0.057  (cbbT1/NC) | Protein folding |
