# Supplementary material for: Revealing the Functions of the Transketolase Enzyme Isoforms in Rhodopseudomonas palustris Using a Systems Biology Approach
Source: PLoS One. 2011 Dec 8;6(12):e28329. doi: 10.1371/journal.pone.0028329 (PMC3234253; doi:10.1371/journal.pone.0028329)
Supplement: Table S5 — Primers for qPCR of photosynthetic genes. (DOC) [file pone.0028329.s007.doc]

**Table S5. Primers for qPCR of photosynthetic genes.**

| **Gene name** | **Common**  **name** | **For/Rev** | **Primer sequence (5’→3’)** |
| --- | --- | --- | --- |
| RPA1491 | *pucBe* | For | CAACAAGGTCTGGCCGACCG |
|  |  | Rev | GCGCCGAAGATGCGCGT |
| RPA1492 | *pucAe* | For | TTTGGACTGTTGTGAAGCCGA |
|  |  | Rev | CGAAGTGCACCAGGATCGC |
| RPA4291 | *pucBb* | For | GATCGCGGAATCGGAAGAGC |
|  |  | Rev | GCCAGGAAGTGAGCGACGA |
| RPA4292 | *pucAb* | For | GGCAGCGTCACCGTCATCG |
|  |  | Rev | GGTGGCGCCGTTCCAGTACT |
| RPA1493 | *pucC* | For | ATCGCGAGCGCCATCATCT |
|  |  | Rev | GACGACGGCAGAGCCCTGG |
| RPA1525 | *pufB* | For | CGAAGCGGAAGCCAAGGAATT |
|  |  | Rev | GGCGAGGATGTGAGCGACA |
| RPA3013 | *pucBd* | For | CTGACCATCGCGGAATCGG |
|  |  | Rev | AGGAAATGCGCCACGATCG |
