# Supplementary material for: Revealing the Functions of the Transketolase Enzyme Isoforms in Rhodopseudomonas palustris Using a Systems Biology Approach
Source: PLoS One. 2011 Dec 8;6(12):e28329. doi: 10.1371/journal.pone.0028329 (PMC3234253; doi:10.1371/journal.pone.0028329)
Supplement: Materials and Methods S1 — (DOC) [file pone.0028329.s008.doc]

**Supporting Information**

**Supplemental Materials and Methods**

Microarray Data Analysis

The microarray slides were immobilized, prehybridized, hybridized and washed as indicated in the accompanying protocol (Agilent Technologies). The microarray data were analyzed with GeneSpring GX 7.3 software. The customized *R. palustris* microarray slides contained triplicate spots for each gene. The data comprising cbbT1, cbbT2, and NC strains were normalized in GeneSpring using the recommended normalization methods: (a) Data transformation: Set measurements to less than 0.01. (b) Per chip: Normalize to 75th percentile. (c) Per Gene: Normalize to median. Genes with a ≧1.5-fold change in expression were considered to be significantly expressed. Cluster analysis was employed to further differentiate the expression patterns among the four strains. Hierarchical clustering was performed using a smooth correlation coefficient with a default separation ratio and distances.

**Bioinformatics Analysis**

All genes significantly expressed as revealed by the microarray data were categorized and distributed according to biological function, association with cellular components, and molecular function for the purposes of functional annotation. This categorization was based on the Gene Ontology terms found in Blast2GO. To distinguish the over-represented protein classes among the identified genes an enrichment analysis was employed with Fisher’s test of *p*-value < 0.01 using Blast2GO.
